# Ethnopharmacological Studies for the Development of New Drugs

**DOI:** 10.1155/2018/7593296

**Published:** 2018-01-31

**Authors:** José Carlos Tavares Carvalho, Caio Pinho Fernandes, Jesus Rafael Rodriguez Amado, Andrés Navarrete, Lucindo José Quintans-Júnior

**Affiliations:** ^1^Laboratório de Pesquisa em Fármacos, Departamento de Ciências Biológicas e da Saúde, Universidade Federal do Amapá, Macapá, AP, Brazil; ^2^Laboratório de Nanobiotecnologia Fitofarmacêutica, Universidade Federal do Amapá, Macapá, AP, Brazil; ^3^Facultad de Farmácia, Universidad de Oriente, Santiago de Cuba, Cuba; ^4^Laboratório de Farmacologia de Productos Naturales, Departamento de Farmácia, Facultad de Química, Universidad Nacional Autónoma de México, Ciudad de México, Mexico; ^5^Laboratório de Neurociências e Ensaios Farmacológicos, Departamento de Fisiologia, Centro de Ciências Biológicas e da Saúde, Universidade Federal de Sergipe, São Cristóvão, SE, Brazil

Ethnopharmacology is an important discipline for the development of new medicines that are of extreme importance for the conservation and enhancement of the traditional use of natural products and undoubtedly is one of the main sources faster for the rational development of new medicines. There are several examples of drugs that have been discovered from ethnopharmacologic studies. Therefore, in this context, this special issue aimed to present important studies carried out by researchers from all the areas that explore and dedicate themselves to the following themes, which were submitted for publication in this journal.

For this special publication, 43 manuscripts from various laboratories from all continents of the world were received, with the following topics: ethnopharmacy, ethnopharmacology and ethnobotanical studies, ethnomedicine and bioactive compounds from animals and plants, pharmaceutical technology with natural products, clinical application of natural products in different communities, predictions and in silico studies with natural products, and alternative biological assay for determination of natural product activity.

After careful analysis by several ad doc referees of renowned universities, 11 manuscripts were accepted for publication, standing out for the quality presented and the high possibility of impact in the field of ethnopharmacology.

In the paper “Optimized-SopungSunkiwon, a Herbal Formula, Attenuates A*β* Oligomer-Induced Neurotoxicity in Alzheimer's Disease Models,” it was confirmed by in vivo data that oral administration of Optimized-SopungSunkiwon (OSS) for 14 days attenuated memory impairments and neuronal cell death by modulating gliosis, glutathione depletion, and synaptic damage in the mouse hippocampus induced by A*β*O.

The manuscript “EGHB010, a Standardized Extract of Paeoniae Radix and Glycyrrhizae Radix, Inhibits VEGF-Induced Tube Formation In Vitro and Retinal Vascular Leakage and Choroidal Neovascularization In Vivo” demonstrated that the EGHB010 that is a hot water extract of the rhizome mixture of* Paeonia lactiflora* Pallas and* Glycyrrhiza uralensis* Fisch and in choroidal neovascularization (CNV) area was significantly lower in EGHB010-treated rats than in vehicle-treated rats. These results suggest that EGHB010 is a potent antiangiogenic agent. Thus, the oral administration of EGHB010 may have a beneficial effect in the treatment of vascular leakage and CNV in patients with age-related macular degeneration.

The psychotropic effects of an alcoholic extract from the leaves of* Albizia zygia* (Leguminosae-Mimosoideae) were evaluated and the hydroethanolic extract of* Albizia zygia* exhibited an antipsychotic-like activity in mice. Motor side effects are only likely to develop at higher doses of the extract. The extract does not possess any significant antidepressant effects.

The potential wound healing activities of* Oxytropis falcate* gel (OFG) was demonstrated in the manuscript “Therapeutic Effect and Mechanism of* Oxytropis falcata* Gel on Deep Second-Degree Burn in Rats,” and the mechanism may be related to the increase of biosynthesis and the release of EGF and CD34 and the decreasing p38 and IL-1*β* levels.

In the study “A Molecular Basis for the Inhibition of Transient Receptor Potential Vanilloid Type 1 by Gomisin A,” it has been demonstrated that the double mutation of Y453 and N467 significantly attenuated inhibitory effects by gomisin A. In summary, it revealed the molecular basis for the interaction between TRPV1 and gomisin A and provided a novel potent interaction ligand.

In the paper “Antcin-H Isolated from* Antrodia cinnamomea* Inhibits Renal Cancer Cell Invasion Partly through Inactivation of FAK-ERK-C/EBP-*β*/c-Fos-MMP-7 Pathways,” luciferase reporter assay showed that antcin-H repressed the MMP-7 promoter activity, in parallel to inhibiting c-Fos/AP-1 and C/EBP-*β* transactivation abilities. Moreover, antcin-H suppressed the activity of ERK1/2 and decreased the binding capacity of C/EBP-*β* and c-Fos on the upstream/enhancer region of the MMP-7 promoter. Overall, this study demonstrated that the anti-invasive effect of antcin-H in human renal carcinoma 786-0 cells might be at least in part by abrogating focal adhesion complex and lamellipodium formation through inhibiting the Src/FAK-paxillin signaling pathways and decreasing MMP-7 expression through suppressing the ERK1/2-AP-1/c-Fos and C/EBP-*β* signaling axis. Thus, it was proven that the evidence that antcin-H may be an active component existed in* A. cinnamomea* with anticancer effect.

In the paper “Tang-Luo-Ning, a Traditional Chinese Medicine, Inhibits Endoplasmic Reticulum Stress-Induced Apoptosis of Schwann Cells under High Glucose Environment,” the results showed that TLN attenuated apoptosis by decreasing Ca2+ level in SCs and maintaining ER morphology. TLN could decrease downstream proteins of CHOP including GADD34 and Ero1*α* while increasing P-eIF2*α* as well as decreasing the upstream proteins of CHOP including P-IRE1*α*/IRE1*α* and XBP-1, thereby reducing ER stress-induced apoptosis.

Flavonoid composition and biological activities of ethanol extracts of* Caryocar coriaceum* Wittm, a native plant from Caatinga Biome, Brazil, were a theme presented that demonstrated that the extracts present antileishmanial activity and low toxicity on murine macrophages and erythrocytes. Therefore, these results suggest a potential for the application of* C. coriaceum* fruit's ethanol extracts in the treatment of dermatophyte fungi and leishmaniasis, probably due to the presence of active flavonoids in both extracts.

In the manuscript “A New Technique Using Low Volumes: A New Technique to Assess the Molluscicidal Activity Using Low Volumes,” a technique that was presented to assess the toxic effect has been proven to be a useful tool to detect the lethal and sublethal effect, which could be used as a new evaluation protocol. This data was confirmed through treatment with plant extract and exhibiting IC_50_ values similar to three methodologies.

Renal pathological changes were examined by the staining of HE, Masson, and PASM. Expressions and distributions of fibronectin (FN), laminin (LN), light chain 3 (LC3), and Beclin-1 were observed by immunohistochemistry, in the paper “Sinomenine Hydrochloride Attenuates Renal Fibrosis by Inhibiting Excessive Autophagy Induced by Adriamycin: An Experimental Study.” SINHCl ameliorates proteinuria; meanwhile, it attenuates the renal pathological changes in adriamycin-induced rats and also attenuates renal fibrosis and excessive autophagy by reducing the expression of FN, LN, LC3, and Beclin-1. SIN-HCl attenuates renal fibrosis by inhibiting excessive autophagy induced by adriamycin and upregulating the basal autophagy.

In the paper “Pharmacological Effect of* Caulophyllum robustum* on Collagen-Induced Arthritis and Regulation of Nitric Oxide, NF-*κ*B, and Proinflammatory Cytokines In Vivo and In Vitro,” the authors showed that, compared with the model group, CRME significantly improved symptoms of the arthritis index, limb swelling, and histological findings by decreasing synovial membrane damage, the extent of inflammatory cell infiltration, and the expansion of capillaries in CIA mice. The results also showed that CRME could reduce the levels of IL-1, IL-6, TNF-*α*, and PGE2 and inhibit the expression of NF-*κ*B p65. All these results indicated the anti-inflammatory efficacy of CRME as a novel botanical extraction for the treatment of RA.

